# Knowledge Discovery and interactive Data Mining in Bioinformatics - State-of-the-Art, future challenges and research directions

**DOI:** 10.1186/1471-2105-15-S6-I1

**Published:** 2014-05-16

**Authors:** Andreas Holzinger, Matthias Dehmer, Igor Jurisica

**Affiliations:** 1 Research Unit Human-Computer Interaction, Austrian IBM Watson Think Group, Institute for Medical Informatics, Statistics & Documentation, Medical University Graz, Austria; 2 Institute of Information Systems and Computer Media, Graz University of Technology, Austria; 3 Institute for Bioinformatics and Translational Research, UMIT Tyrol, Austria; 4 Departments of Medical Biophysics and Computer Science, University of Toronto, Ontario, Canada; 5 Princess Margaret Cancer Centre and Techna Institute for the Advancement of Technology for Health, University Health Network, IBM Life Sciences Discovery Centre, Ontario, Canada

**Keywords:** Knowledge Discovery, Interactive Data Mining, Bioinformatics, Biomedical Informatics, Data intensive Science

## 

Computers are incredibly fast, accurate, and stupid.

Human beings are incredibly slow, inaccurate, and brilliant.

Together they are powerful beyond imagination

(Einstein never said that [1]).

## Background

The life sciences, biomedicine and health care are increasingly turning into a data intensive science [2-4]. Particularly in bioinformatics and computational biology we face not only increased volume and a diversity of highly complex, multi-dimensional and often weakly-structured and noisy data [5-8], but also the growing need for integrative analysis and modeling [9-14].

**Figure 1:**
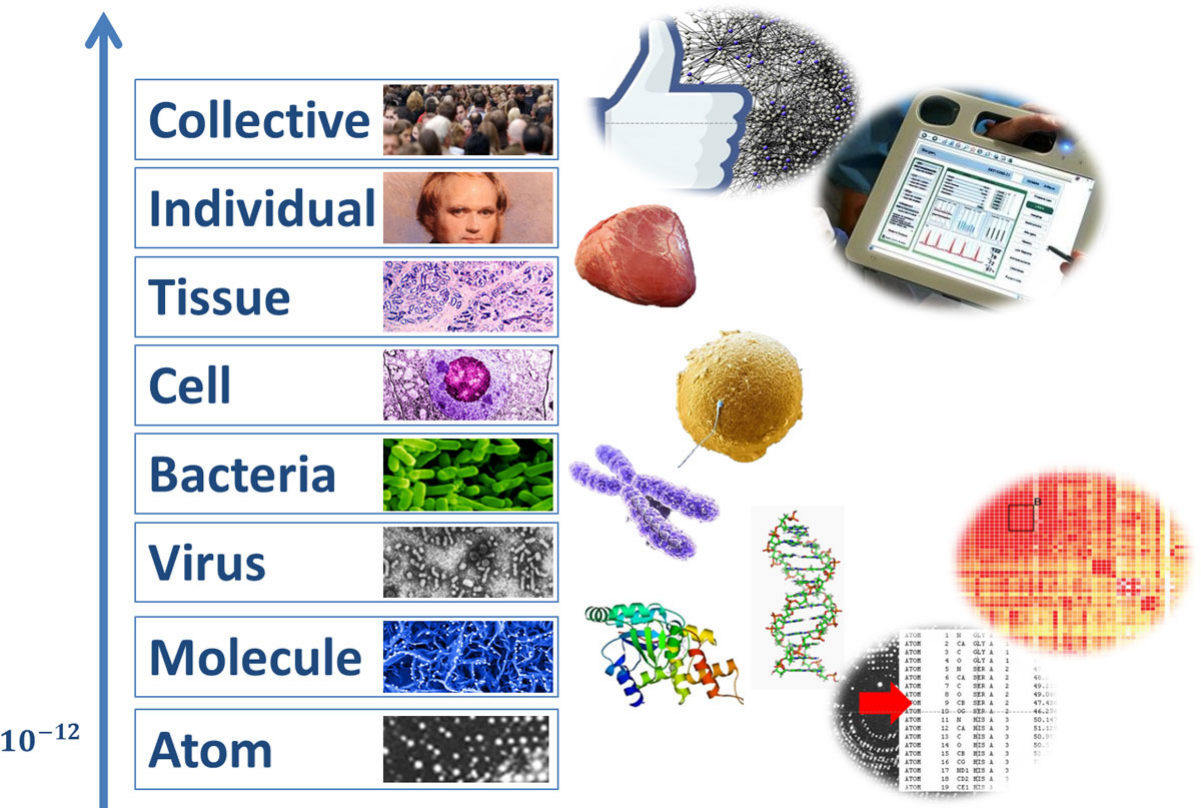
**The trend towards personalized and molecular medicine brings together data from very different sources**.

Due to the increasing trend towards personalized and precision medicine (P4 medicine: Predictive, Preventive, Participatory, Personalized [15]), biomedical data today results from various sources in different structural dimensions, ranging from the microscopic world, and in particular from the omics world (e.g., from genomics, proteomics, metabolomics, lipidomics, transcriptomics, epigenetics, microbiomics, fluxomics, phenomics, etc.) to the macroscopic world (e.g., disease spreading data of populations in public health informatics), see Figure 1[16]. Just for rapid orientation in terms of size: the Glucose molecule has a size of $900\;pm=900\times 10^{-12}m$ and the Carbon atom approx. 300 pm. A hepatitis virus is relatively large with $\text{45}\;nm=45\times 10^{-9}m$ and the X-Chromosome much bigger with $\text{7}\;\mu m=7\times 10^{-6}m$. We produce most of the "Big Data" in the omics world, we estimate many Terabytes ($1\;\text{TB}=1\times 10^{12}\;\text{Byte}=1000\text{ G}\;\text{Byte}$) of genomics data in each individual, consequently, the fusion of these with Petabytes of proteomics data for personalized medicine results in Exabytes of data ($\text{1}\;\text{EB}=1\times 10^{18}\;\text{Byte}$). Last but not least, this "natural" data is then fused together with "produced" data, e.g., the unstructured information (text) in the patient records, wellness data, the data from physiological sensors, laboratory data etc. - these data are also rapidly increasing in size and complexity. Besides the problem of heterogeneous and distributed data, we are confronted with noisy, missing and inconsistent data. This leaves a large gap between the available "dirty" data [17] and the machinery to effectively process the data for the application purposes; moreover, the procedures of data integration and information extraction may themselves introduce errors and artifacts in the data [18].

Although, one may argue that "Big Data" is a buzz word, systematic and comprehensive exploration of all these data is often seen as the *fourth paradigm *in the investigation of nature - after empiricism, theory and computation [19], and provides a mechanism for data driven hypotheses generation, optimized experiment planning, precision medicine and evidence-based medicine.

The challenge is not only to extract meaningful information from this data, but to gain knowledge, to discover previously unknown insight, look for patterns, and to make sense of the data [20], [21]. Many different approaches, including statistical and graph theoretical methods, data mining, and machine learning methods, have been applied in the past - however with partly unsatisfactory success [22,23] especially in terms of performance [24].

The grand challenge is to make data useful to and useable by the end user [25]. Maybe, the key challenge is *interaction*, due to the fact that it is the human end user who possesses the problem solving intelligence [26], hence the ability to ask intelligent questions about the data. The problem in the life sciences is that (biomedical) data models are characterized by significant complexity [27], [28], making manual analysis by the end users difficult and often impossible [29]. At the same time, human experts are able to solve complicated problems sometimes intuitively [30], [31], [32], e.g., often without being able to describe the exact rules or processes used during their analysis and problem solving.

Many advances in powerful computational tools [33], [34] in recent years have been developed by separate communities with different philosophies: Machine learning researchers tend to believe in the power of their statistical methods to identify relevant patterns [35] - mostly automatic, without human intervention [36]; however, the dangers of modelling artefacts grow when end user comprehension and control are diminished [37].

Additionally, mobile, ubiquitous computing and sensors, together with low cost storage, will accelerate this avalanche of data [38], and there will be a danger of drowning in data but starving for knowledge, as Herbert Simon pointed it out 40 years ago: *"A wealth of information creates a poverty of attention and a need to allocate that attention efficiently among the overabundance of information sources that might consume it" *[39].

Consequently, it is a grand challenge to work towards enabling effective human control over powerful machine intelligence by the integration and combination of machine learning methods and advanced visual analytics methods to support insight and decision making [28,40-44].

We envision effectively tackling these challenges by bringing together the best of two worlds: A synergistic combination of theories, methods and approaches from Human-Computer Interaction (HCI) and Knowledge Discovery from Data (KDD). Such approaches need a trans-disciplinary methodology. For example, the understanding of complex structures, such as regulatory networks, is a challenging objective and one that cannot be tackled within a single, isolated discipline [45]. Also, advances in network-based methods are enabled by novel applications. This relates to the exploration of methods and measures [46,47] to investigate global and local structural properties of complex networks or to study their interrelations [48-50]. While the relevant literature of the last decades has portrayed the definition of infinitely many network measures and methods as a relatively simply undertaking; overall, understanding this complex mathematical apparatus has turned out to be very complicated [51,52].

There is no doubt about the usefulness of such techniques in general. However, this branch of science somewhat failed to demonstrate the usefulness and interpretability of the underlying mathematical apparatus. In fact, while this development led to a vast amount of network measures/methods, exploring their structural interpretation and meaning has been often overlooked. This calls for generating more results to interpret the measures/methods more properly.

## Knowledge Discovery process

The traditional method of turning data into knowledge relied on manual analysis and interpretation by a domain expert in order to find useful patterns in data for decision support. An early example from medical diagnostics includes the work by Reeder & Felson (1977) [53]. Today, far beyond pattern recognition, this process has been given a variety of names, including: data mining, knowledge extraction, information discovery, information harvesting, data archaeology, and data pattern processing [54]. In the classic work by Fayyad et al. (1996), [55], this process is described by different steps starting from data selection, pre-processing, data transforming, data mining and interpretation. In this definition, Data Mining is actually a subset of Knowledge Discovery, and although the original notion was Knowledge Discovery in Databases (KDD), today, in order to emphasize that Data Mining is an important subset of the knowledge discovery process, the current most used notion is Knowledge Discovery and Data Mining (KDD). It is important to note that KDD can be seen as a *process *and encompasses the complete value added chain from the very physical side of data to the very human side of knowledge, the latter defined from a cognitive point of view: knowledge as a set of expectations [56]. We further extend the original definition by Fayyad et al. (1996) by *interaction *and include the human-into-the-loop. Interaction, communication and sensemaking are core topics in Human-Computer Interaction (HCI) [25,57-61], consequently, a novel approach is to combine HCI & KDD [8,44].

**Figure 2:**
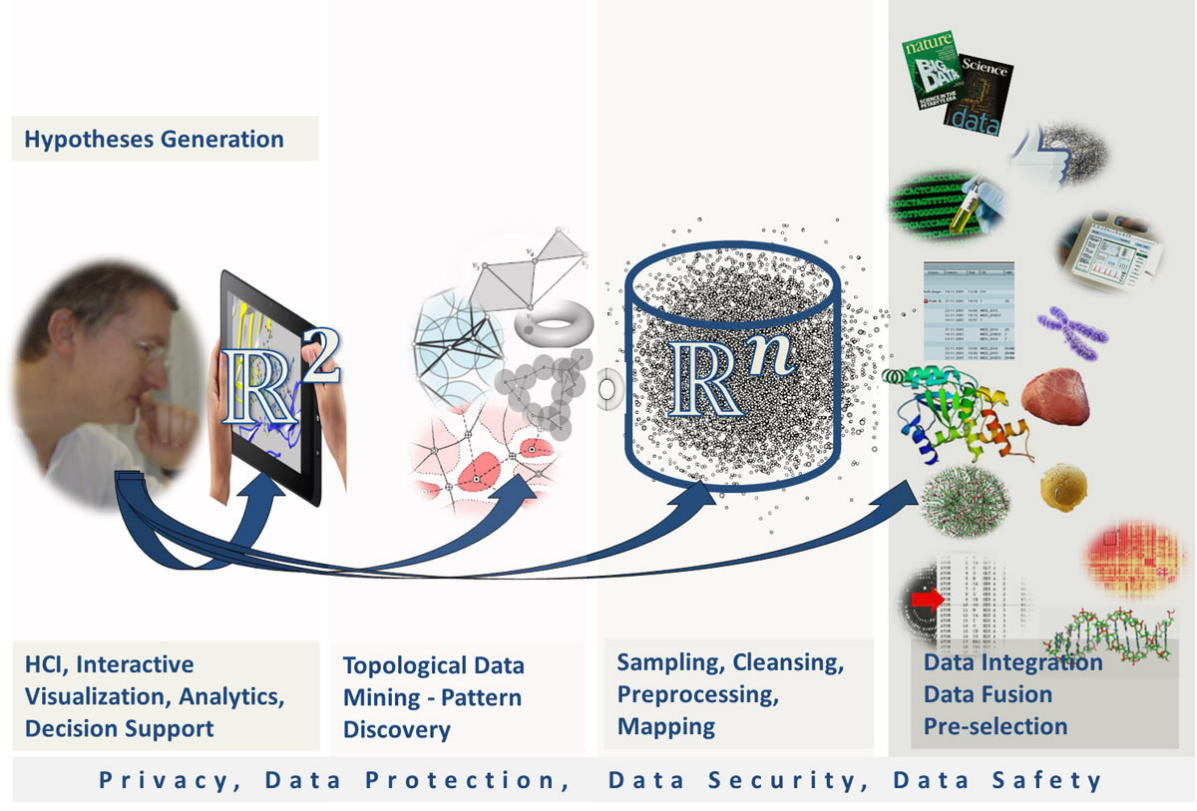
**The knowledge discovery process in the life sciences**.

The central premise of HCI-KDD is to *enable *end users interactively to *find and characterize *previously unknown and potentially useful and usable information. It may be defined in the classical sense as the process of identifying novel data patterns, with the goal of understanding these patterns. The domain expert in Figure 2 possesses explicit domain knowledge and by enabling them to interactively explore the data sets, they may be able to identify, extract and understand useful information, to gain new, and previously unknown knowledge [21].

KDD historically builds on three fields: machine learning; databases and artificial intelligence to design and develop tools and frameworks that let the end users gain insight into the nature of massive data sets [54], [24], [62].

## Future research directions

Figure 2 illustrates the complete knowledge discovery process, and we will use this "big picture" for the description of some problems and challenges - starting (in this Figure) from right to left - from the computer to the human - segmenting it into four large areas:

### Area 1: Interactive data integration, data fusion and pre-selection of data sets

Many different biological species (humans, animals, bacteria, virus, plants, ...) deliver large amounts of data, together with the enormous complexity of medicine per se [42] and the limited computational power in comparison of the complexity of life (and the natural limitations of the Von-Neumann architecture) these pose a lot of problems, which can be divided into three categories:

• Heterogeneous data sources (need for data fusion);

• Complexity of the data (high-dimensionality);

• Noisy, uncertain data, dirty data, the discrepancy between data-information-knowledge (various definitions), Big data sets (when is data big? when manual handling of the data is impossible) [24].

In comparison to research systems, commercially available information systems have only limited data fusion capabilities, if any at all [63]. It is a huge challenge to integrate and fuse the biological data together with classical patient records, physiological data or medical image data [64], [65]. The issues are so big that there is an own conference series called "data integration in the life sciences" [66].

### Area 2: Interactive sampling, cleansing, preprocessing, mapping

The problem of merging multiple data sets concerning common entities is frequently encountered in KDD, often called the Merge/Purge problem, it is difficult to solve both in scale and accuracy [67]. Cleansing data from impurities is an integral part of every data processing and has led to the development of a broad range of methods to enhance the accuracy and thereby the usability of existing data [68]. Many machine learning algorithms, for example, struggle with high-dimensional data. This has become well known as the curse of dimensionality [69]. A further issue is that most medical data is incomplete, with missing data values, inconsistent value naming conventions, etc. or requires the detection and removal of duplicate data entries [70] - so the central goal of data quality poses a number of problems and challenges [71], [72]. The quality of data finally, influences the quality of information [73].

### Area 3: Interactive advanced data mining methods, pattern discovery

Many data mining methods are designed for collections of objects well-represented in rigid tabular formats. However, besides massive sets of unstructured information and non-standardized information (text) [74-76], we are increasingly confronted with large collections of interrelated objects whose natural representation is in point cloud data or typed graphs [77] (e.g., protein structures, protein interaction networks, etc.).

Advanced data mining approaches include:

1) graph-based data mining [78], [79], [80], [81],

2) entropy-based data mining [47,82], [83-85], and

3) topological data mining [86,87].

We emphasize that these approaches are interdisciplinary and complementary albeit having common goals, and have been proven useful to perform translational research, e.g., [47,82,84,85].

In particular, entropy-based graph analysis is based on using information theory and graph theory. Generally, information theory [88] relates to quantifying information and to investigating communication processes. To translate this concept to graph theory has been intricate. As a result, various graph entropies have been developed but the literature lacks exploring interrelations with other network measures. An example thereof can be found in [47]. Much future research is necessary in this area in the future.

### Area 4: Interactive visualization, HCI, analytics, decision support

Finally, the results gained by the application of sophisticated algorithms in high dimensional spaces in area 3 must be mapped back to ${\mathbb{R}}^{2}$ because humans have difficulties in comprehending higher dimensional data.

We can say that, while our world is highly dimensional mathematically, we can only perceive lower dimensions. This leads to the definition of visualization *as the mapping from the higher into the lower dimensional space*, a process that always suffers the danger of modelling artefacts. Although Visualization is a mature field with a background of several decades, there are still a lot of challenging and open research issues, especially in the context of interactive data mining with application to the biomedical domain. A major issue is the absence of a complete toolset that supports all analysis tasks within a biomedical workflow, including the many steps of data preprocessing [89]. It is very interesting to note that although there are many sophisticated visualization techniques available [90-102], - these are rarely applied in routine applications, especially in business enterprise hospital information systems, where such approaches really could bring benefits to the professionals. An extremely important issue is the limited time, e.g., in average a medical doctor in a public hospital has only five minutes to make a decision [103,104]; This strongly calls for interactive tools. Naive visualization attempts are often ineffective or even actively misleading, due to the fact that the development of effective visualizations is a complex process and requiring a basic understanding of human information-processing and a solid grounding in the existing body of work in the visualization community [105-107].

### Horizontal area: Privacy, data protection, data security, data safety

Whenever we deal with biomedical data issues of privacy, data protection, data security and data safety and the fair use of data are of paramount importance [108], including data accessibility, temporal limits, legal restrictions (such as in situations where copyright or patents may be relevant), confidentiality and data provenance. We face a range of research challenges in developing data mining methods to properly handle these complex restrictions.

## Additional aspects to consider

Some additional aspects to consider include:

### Cross-disciplinary cooperation with domain experts

Building a project consortium comprising of experts with complementary expertise but common interests is a success factor in each project. Bringing together domain experts from diverse areas in a cross-disciplinary manner is a challenge to stimulate fresh ideas and encouraging multi-disciplinary work [109]. For example, the application of principles from HCI to data-driven projects in biomedical contexts has been lacking and has been receiving increasing attention in recent years [59], [110]. In the life sciences domain, experts are both data producers and end users at the same time, knowledge engineers and analysts help to organize, integrate, visualize, analyze and evaluate the data. For example, in "systems biology" intertwining these two may lead to improving both the models and the experimental results. In such complex domains as in biomedicine, we need experts who understand the domain, the problem, and the data sets, hence the context [111].

### Interpretability

As we broaden workflows for data mining, we have to expand metrics used to evaluate our results. It is no longer sufficient to focus on performance metrics, such as ROC [112], accuracy, precision and recall (although precision and recall still are *the *measures in data mining [113]), one must also consider how non-functional requirements are satisfied, such as interpretability. In the biomedical domain, where it is necessary to explain or justify the results of a decision, data mining alone is definitely irrelevant: It is necessary to produce results that are explainable to others. In a SIAM conference in 2007 an interesting panel was held, where the panelists including Christos Faloutsos (Carnegie Mellon University), Jerry Friedman (Stanford University), Ajay Royyuru (IBM Research), and Mehran Sahami (Google Research), together with the moderator Haym Hirsh (Rutgers University), formulated a couple of interesting questions, which are very relevant up to the present [23], for example: How can we quantitatively and qualitatively measure interpretability? Similar to the concepts of interest or beauty [114], interpretability is in the eye of the beholder and possibly dependent on the previous knowledge and the level of expertise of the decision maker [115], consequently, we need adaptive tools to satisfy both novices and experts.

### Computing resources

As our computing machinery evolves, from large main-frame servers to multi-core CPU/GPU clusters we need to optimize data mining algorithms, processes and workflows to best fit the environment. The potential of so-called On-Demand Hardware along with the Software as a Service (SAAS) paradigm [116] can no longer be denied, and there are several examples yet of Cloud Computing approaches, e.g. in drug discovery research, medical imaging and applications for doctors in rural areas [117-119]. However, much data in biomedicine and healthcare has strict privacy requirements and therefore privacy, security safety and data protection issues are of enormous importance with such future approaches. Major internet companies offer already such services for data-intensive computing and a similar strategy led to the developing of large computing grids for massive data analysis, such as IBM's World Community Grid (http://www.worldcommunitygrid.org), [120].

### Benchmarking against Gold-Standards

To measure the quality of data mining approaches, the production of benchmarks it very important. These data sets can be used as so-called gold-standards (e.g., [121-123], which allow us to compare results across competing methods and are thus important for information quality issues [124,125].

### Reproducibility

A big general issue among our modern research communities is that rarely one can reproduce the results of other researchers. Often it is not possible to verify and to replicate experiments, which is the case for example in classical non-computing experimental sciences [126]. One of the major issues is "sloppiness in data handling" and the resulting exponentially growing retraction of papers [127]. So, a mega challenge is in ensuring that results can be replicated from other groups at other places.

### Embedded data mining

Whilst existing research has shown the value of data-driven science, we need to further integrate knowledge discovery and visualization pipelines into biological and biomedical and especially clinical workflows to take full advantage of their potential [23].

### Complexity of data analysis methods

Deciding which method is the most suitable for solving a particular data analysis problem is often critical as the interdependencies make the selection non-linear [128]. Hence to perform data analysis efficiently, a deep understanding of the underlying mathematical apparatus is necessary.

## Conclusion

We are just at the beginning of a turning point towards data intensive life sciences, which entails many challenges and future research directions. Within this overview we have highlighted only a few issues. Summarizing, we may say that the grand challenge is in building frameworks for enabling domain experts to interactively deal with their data sets in order to "ask questions" about the data, for example: "Show me similarities/differences/anomalies of data set × and data set Y", hence the discovery of novel, previously unknown patterns in complex data. Which mathematical framework should we use? One challenge is that such a framework must be usable for domain experts without prior training in mathematics or computational sciences. We need machine intelligence to deal with the flood of data, but at the same time we must acknowledge that humans possess certain problem solving and cognition abilities, which are far beyond computation. A possible solution is in the cross-disciplinary combination of aspects of the better of two worlds: Human-Computer Interaction (HCI) and Knowledge Discovery from Data (KDD). A proverb attributed perhaps incorrectly to Albert Einstein illustrates this perfectly: "Computers are incredibly fast, accurate, but stupid. Humans are incredibly slow, inaccurate, but brilliant. Together they may be powerful beyond imagination".

## Authors' information

**Andreas Holzinger **is head of the Research Unit Human-Computer Interaction, Institute for Medical Informatics at the Medical University Graz, Lead at the HCI-KDD network, head of the first Austrian IBM Watson Think Group, Associate Professor of Applied Informatics at the Faculty of Computer Science, Institute of Information Systems and Computer Media and Lecturer at the Institute of Genomics and Bioinformatics at Graz University of Technology. He serves as consultant for the Canadian, Swiss, French and Dutch Government, for the German Excellence Initiative and as national expert in the European Commission (Lisbon Delegate 2000). Andreas, born 1963, started as an apprentice in IT in 1978; while working as an industrial engineer, he resumed a parallel second-chance education, finished his PhD in Cognitive Science in 1997 and completed his second doctorate (Habilitation) in Computer Science in 2003. Since 1999 participation in leading positions in 30+ R&D multi-national projects, budget 3+ MEUR; 300+ publications, >4000+ citations. Andreas was Visiting Professor in Berlin, Innsbruck, Vienna, London, and Aachen. He is passionate on bringing together Human-Computer Interaction (HCI) and Knowledge Discovery/Data Mining (KDD), with the goal of supporting human intelligence with machine intelligence - to discover new, previously unknown insights into complex biomedical data. http://www.hci4all.at

**Matthias Dehmer **is currently head of the Division for Bioinformatics and Translational Research at UMIT, Austria and a professor of discrete mathematics. He studied mathematics and computer science at the University of Siegen (Germany) where he graduated in 1998. Between 1998 and 2002, he held positions as a mathematical researcher and a business consultant in industry. He joined in 2002 the Department of Computer Science at Darmstadt University of Technology and obtained a PhD in computer science. From 2005 to 2008, he held several research positions at the University of Rostock (Germany), Vienna Bio Center (Austria), Vienna Technical University (Austria) and University of Coimbra (Portugal). Finally, he obtained his habilitation in applied discrete mathematics from the Vienna University of Technology. He has been the head of the Division for Bioinformatics and Translational Research at UMIT, Austria. He has published over 160 publications in applied mathematics and computer science. Moreover, he is an editor of the book series "Quantitative and Network Biology", Wiley-VCH. He organized and co-organized several international scientific conferences and workshops in USA. Also, he recently got a member of the editorial board of Scientific Reports (Nature) and PLoS ONE. http://www.dehmer.org

**Igor Jurisica **isTier I Canada Research Chair in Integrative Cancer Informatics, is a Senior Scientist at Princess Margaret Cancer Centre, Professor at the University of Toronto and Visiting Scientists at IBM's Centre for Advanced Studies. He is also an Adjunct Professor at the School of Computing, Department of Pathology and Molecular Medicine Queen's U and Department of Computer Science and Engineering at York University. Igor's research focuses on integrative computational biology and the representation, analysis and visualization of high-dimensional data to identify prognostic/predictive signatures, drug mechanism of action and in silico re-positioning of drugs. Interests include comparative analysis for mining different integrated data sets (e.g., protein-protein interactions, high-dimensional cancer data, and high-throughput screens for protein crystallization). http://www.cs.toronto.edu/~juris.

## Competing interests

All authors declare that they have no competing interests.

## Declarations

Publication for this article has been funded by the Research Unit hci4all.at

This article has been published as part of *BMC Bioinformatics *Volume 15 Supplement 6, 2014: Knowledge Discovery and Interactive Data Mining in Bioinformatics. The full contents of the supplement are available online at http://www.biomedcentral.com/bmcbioinformatics/supplements/15/S6.
